# Four overlooked errors in ROC analysis: how to prevent and avoid

**DOI:** 10.1136/bmjebm-2024-113078

**Published:** 2024-09-19

**Authors:** Zhuoqiao He, Qingying Zhang, Manshu Song, Xuerui Tan, Wei Wang

**Affiliations:** 1 Department of Cardiology, First Affiliated Hospital of Shantou University Medical College, Shantou, Guangdong, China; 2 Centre for Precision Health, School of Medical and Health Sciences, Edith Cowan University, Perth, Western Australia, Australia; 3 Department of Preventive Medicine, Shantou University Medical College, Shantou, China; 4 Clinical Research Centre, First Affiliated Hospital of Shantou University Medical College, Shantou, Guangdong, China; 5 Human Phenome institute, Guangdong Engineering Research Center of Human Phenome, Chemistry and Chemical Engineering Guangdong Laboratory, Shantou, Guangdong, China; 6 Institute for Glycome Study, Shantou University Medical College, Shantou, Guangdong, China; 7 School of Public Health, Shandong First Medical University & Shandong Academy of Medical Sciences, Tai’an, Shandong, China

**Keywords:** Diagnosis, Methods, Evidence-Based Practice

## Introduction

 Diagnostic tests are frequently applied within clinical practice to assist with disease diagnosis, differential diagnosis, disease grading and prognosis evaluation. Receiver operating characteristic (ROC) curve analysis is one common approach for analysing discriminative performance of a diagnostic test, where it can determine the optimal cut-off value with the best diagnostic performance.^1^ However, as a majority of clinicians are non-statisticians, several errors have been observed in clinical research when applying ROC curves. These errors may be misleading in the selection of diagnostic tests and disease diagnosis, thus adding to patient burden. To address these errors, clinicians do not need a deep understanding of the intricate mathematical formulas of ROC analysis, but should develop basic knowledge and skills to prevent or avoid commonly overlooked mistakes. This article aims to guide clinicians to avoid common pitfalls in ROC analysis.

## Basic knowledge of ROC curve

The ROC curve is a graphical representation for evaluating the performance of a binary classification model or diagnostic test, plotted in a 1×1 square with: the vertical axis representing sensitivity or true positive rate (TPR), defined as the number of true positives divided by the total number of positive cases^2^; while the horizontal axis represents 1−specificity rate or false positive rate (FPR), calculated as the ratio of false positives to the total number of negative cases.^2 3^ In diagnostic tests with quantitative or multiclass results, each possible test value can serve as a potential cut-off point. ROC analysis enables the evaluation of different cut-off points, calculating sensitivity and specificity for each one.^4^ Additionally, ROC curve has been characterised as ‘a two-dimensional depiction of classifier performance’.^3^ Classifiers are typically compared according to the area under the curve (AUC), which ranges between 0 and 1.0, representing the area of the unit square. The practical lower limit for the AUC of a classifier is 0.5.^5^ ROC analysis primarily focuses on comparing AUCs and determining cut-off values in diagnostics. Four overlooked errors include three in AUC comparison and one in cut-off selection.

## General operations of ROC analysis

**Figure 1:**
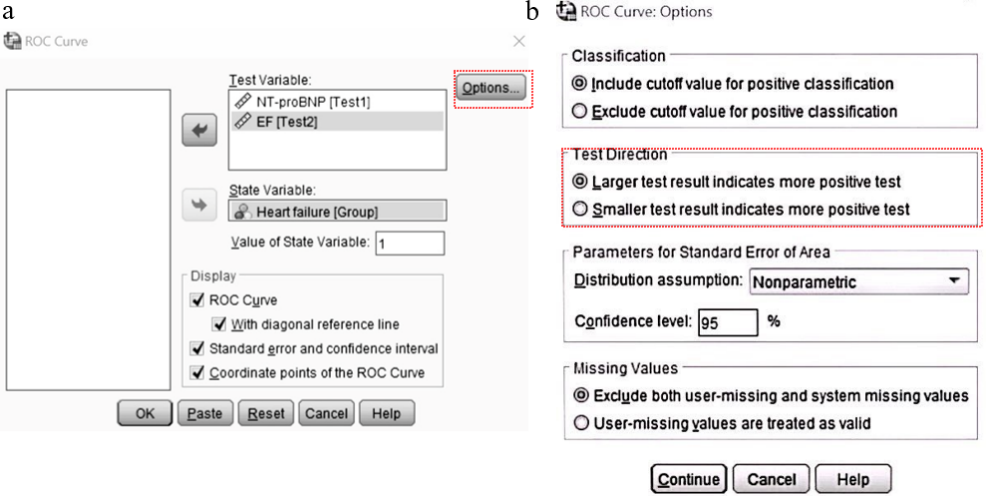
(a) The ROC analysis operation box in SPSS. (b) The box for the option of ‘Test Direction’ in SPSS. EF, ejection fraction; NT-proBNP, N-terminal pro-B-type natriuretic peptide; ROC, receiver operating characteristic.

Below is an example to guide clinicians in performing ROC analysis using SPSS V.26.0, a commonly used statistical analysis software. It is established that N-terminal pro-B-type natriuretic peptide (NT-proBNP) and ejection fraction (EF) are used for the diagnosis of heart failure (HF). When comparing their diagnostic performances using ROC analysis, NT-proBNP and EF are considered ‘test variable’, whereby the presence ‘1’ or absence ‘0’ of HF is considered the ‘state variable’. The ‘value of state variable’ indicates which category should be considered positive, with the presence of HF generally considered a positive state (‘1’ in this example, figure 1a). To proceed, ‘options’ can be clicked to select the ‘test direction’ (figure 1b). If higher test results increase the likelihood of HF, choose ‘larger test result indicates more positive test’. Conversely, if lower test results suggest a higher likelihood of HF, select ‘smaller test result indicates more positive test’.^6^ Accordingly, for NT-proBNP, choose ‘larger test result indicates more positive test’, and for EF, select ‘smaller test result indicates more positive test’.

## Error 1：AUC<0.5

**Figure 2:**
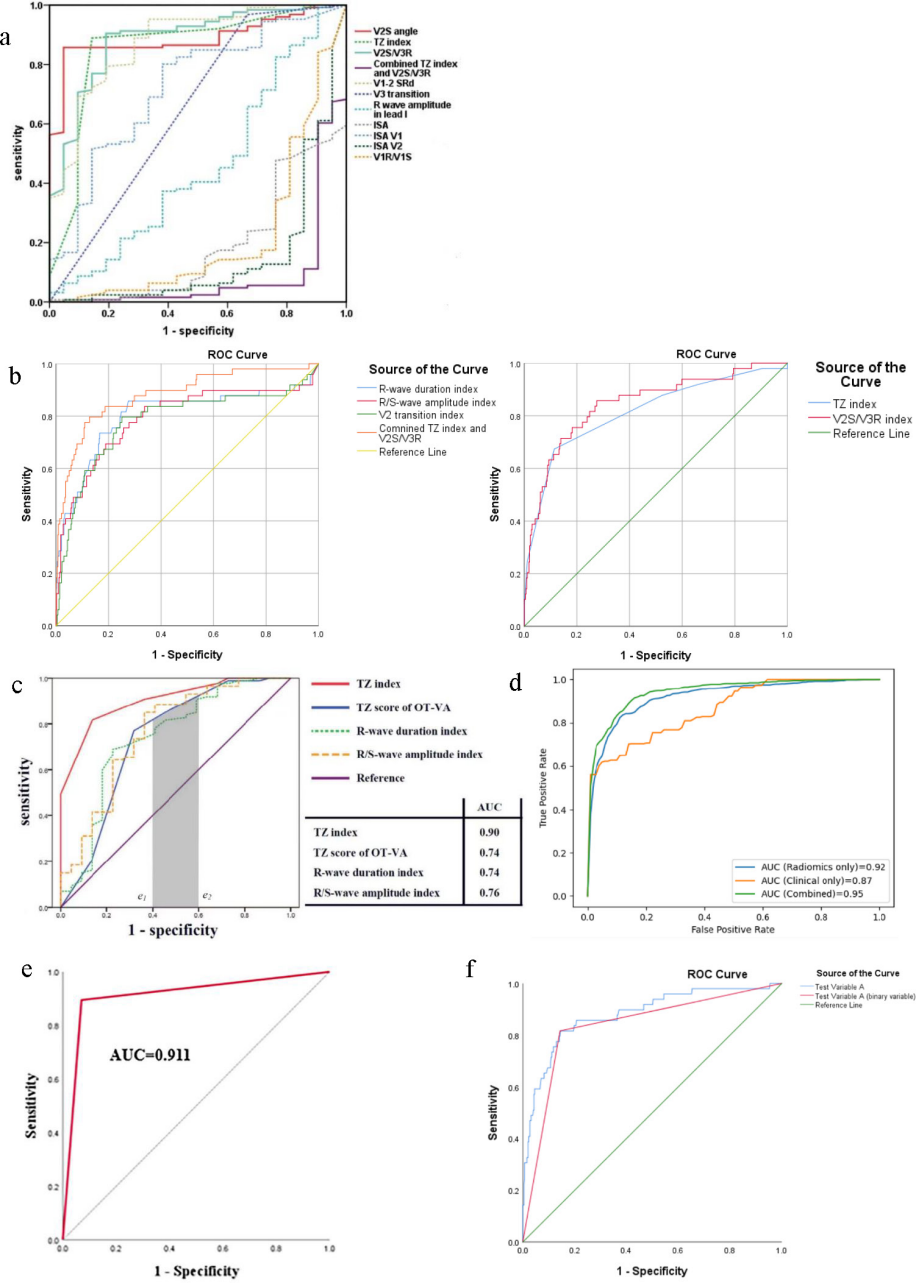
(a) The ROC curves of ECG algorithms from Qiu *et al*.^7^ (b) Published data based on our previous study^8 20^ is used to compare different ECG algorithms, including the TZ Index and Combined TZ Index and V2S/V3R. After changing the ‘test direction’ for the Combined TZ Index and V2S/V3R, the ROC curves for all ECG algorithms appear above the reference line in two distinct graphs. (c) The ROC curves of four ECG algorithms in Yoshida *et al*’s study^12^ are modified, where TZ score of OT-VA and R-wave duration index have an equal AUC. The grey area represents the partial AUC, which can be compared between these two ROC curves within a specific false positive rate range (e_1_=0.4, e_2_=0.6) (Rightslink License Number 5846520834586). (d) DeLong test can be performed to assess the discriminative power of models. (e) A single cut-off ROC curve in Wang *et al*’s study^19^ (Rightslink License Number 5837510051304). (f) Published data based on previous study^20^ is used to plot ROC curves with the test variable being a continuous variable and a binary variable, respectively. AUC, area under the curve; OT-VA, outflow tract ventricular arrhythmia; ROC, receiver operating characteristic; TZ, Transitional Zone; V2S, V2 S-wave amplitude; V3R, V3 R-wave amplitude.

For clinicians, it is important to recognise that a realistic diagnostic test should have an AUC of at least 0.5, since random guessing (or flipping a coin) produces a diagonal line with an area of 0.5^3^ (ie, the discriminative power of a diagnostic test should be greater than that of tossing a coin). When an ROC curve significantly descends towards the lower right half of the graph, this implies that the diagnostic accuracy of the test is lower than random chance. This could result from an incorrect state value or a wrong test-state association direction for determining a positive test result, which has been selected in the ‘test direction’ section of the ‘ROC curve: options’ (figure 1b).^6^ Clinicians, therefore, should select the ‘test direction’ correctly. For example, one study has compared discriminative performances of several ECG algorithms using ROC analysis, whereby five of the eleven AUC values were smaller than 0.5 (figure 2a).^7^ Among these, taking the Transitional Zone (TZ) Index (AUC>0.5) and Combined TZ Index and V2 S-wave amplitude/V3 R-wave amplitude (Combined Index) (AUC<0.5) as examples, larger values of the TZ Index indicate increasing likelihood of the state value, while smaller values of the Combined Index indicate increasing likelihood of the state value.^7^ As noted above, AUC<0.5 is incorrect, where it should be larger than 0.5 after changing the ‘test direction’ (ie, selecting the ‘larger test results indicate more positive tests’ for the TZ Index, whereas selecting the other one for the Combined Index). This type of error can be remedied (as shown in figure 2b and our previous publication^8^).

## Error 2：intersection curve

The second error related to AUC comparison occurs when two ROC curves intersect. While computation of AUC is a well-established measure of the discriminative power of different diagnostic tests,^9 10^ simply comparing AUC values is only meaningful when two ROC curves do not intersect (ie, one curve is consistently above the other).^11^ If two curves intersect, solely using AUC values to evaluate diagnostic performance is insufficient. In such cases, it is crucial to consider additional metrics such as partial AUC (pAUC), which compute AUC in the area of the ROC space that corresponds to interesting (ie, practically viable or acceptable) values of FPR and TPR. Other important metrics include accuracy (the ratio of correct predictions to total predictions), precision (the ratio of true positives to total positives) and recall (which is equivalent to TPR/sensitivity). As an example, one study comparing the discriminative performance of four ECG algorithms found that although the two AUC values for the TZ score of outflow tract ventricular arrhythmia (OT-VA) and the R-wave duration index were the same (AUC=0.74),^12^ this does not necessarily indicate equivalent diagnostic performance. The TZ Score of OT-VA can be superior in a specific region of the curve (high FPR range), whereas the R-wave duration index may excel in another region (low FPR range). In such scenarios, pAUC, computed as the AUC where e_1_≤FPR≤e_2_ (FPR_1_=e_1_ and FPR_2_=e_2_),^5^ should be presented to provide a more detailed assessment of performance in specific regions of the curve^1 13^ (figure 2c). Further, metrics including accuracy, recall and precision should also be evaluated to provide a comprehensive assessment.^3^ In clinical settings, the choice of a diagnostic test should be tailored to the specific diagnostic scenario. For primary screening among healthy subjects, tests with high sensitivity (high TPR or recall) are preferred. Conversely, for diagnosing suspected patients, tests with high specificity are more appropriate.^14^ When the cost of a false positive is high, such as in cancer diagnosis, a test with high precision ensures that patients identified as having cancer are indeed likely to have it, reducing unnecessary stress and invasive treatments.

## Error 3：comparison between AUCs

The third error in AUC comparison occurs when diagnostic tests have similar AUC values. In such cases, a simple comparison of absolute AUC values may not be sufficient. To make a further comparison, additional statistical tests should be used. For ROC curves derived from the same subjects, DeLong test is appropriate for comparison.^15 16^ For ROC curves derived from two independent sample sets, the Dorfman and Alf method could be used.^17^ However, this critical point has been often overlooked. For example, one study compared discriminative performances among clinical (AUC=0.87), radiomics (AUC=0.92) and combined clinical–radiomics (AUC=0.95) models using ROC analysis (figure 2d). The study concluded that the combined model is superior to the other two based solely on absolute values of AUC.^18^ In cases like this, DeLong test can help assess the statistical significance of differences between the ROC curves, ensuring that even minor differences are evaluated appropriately.

## Error 4：single cut-off ROC curve

The last error identified in this paper involves the occurrence of a single cut-off ROC curve when determining the optimal cut-off value for a test. This type of ROC curve features only one inflection point and two straight lines (figure 2e). When a test variable (diagnostic test) is continuous or involves multiple classes, each possible test value can be considered a potential cut-off point, determining the corresponding TPR and FPR. The optimal cut-off value is then selected based on specific clinical requirements.^1 3^ However, if a test variable is binary, the ROC curve is sharply shaped by a single cut-off point, with TPR and FPR calculated based on the outcomes of binary classification at that fixed threshold.^3^ For example, one study developed an intrahepatic cholangiocarcinoma (ICC) scoring system (ie, −2.474−2.554×elevated Alpha-fetoprotein+2.537×elevated CA 19-9+2.451×obscure lesion boundary+3.164×Rim-like hyperenhancement+1.976×wash-out onset within 45s+2.976×marked wash-out within 3 min).^19^ ROC analysis was performed in the study to determine the optimal cut-off value for the score ‘1.322’ (figure 2e).^19^ The ICC score is a continuous variable, but this ROC curve presented a single cut-off shape. To overcome this issue, we use our published data as an example^20^: test variable A, a continuous variable equivalent to ICC score in the above case, yields a curve with the optimal cut-off value shown in figure 2f. However, when we convert test variable A into a binary variable based on this cut-off value and plot ROC curve again, the result is a single cut-off curve (figure 2f, test variable A as binary variable). Therefore, for an ROC curve based on the ICC score presented as a single cut-off curve, it can be speculated that either the ICC score has only two values or that binary classification defined by the optimal cut-off value has been used for plotting. Regardless of the reason, this occurs because a binary variable was used to plot the ROC curve.

## Conclusion

This article identifies four often overlooked errors in statistical analysis within diagnostic medicine during ROC analysis. Errors 1, 2 and 3 can lead to a misleading assessment of the discriminative power of a diagnostic test, while error 4 may result in an incorrect optimal cut-off value. Thus, it is crucial for clinicians to understand these common pitfalls to prevent and avoid these mistakes in their statistical analyses and data presentation in academic publications.
